# Oncoplastic breast surgery versus conventional breast‐conserving surgery: a comparative retrospective study

**DOI:** 10.1111/ans.15245

**Published:** 2019-04-16

**Authors:** Ilmi Behluli, Pol‐Edern Le Renard, Kamila Rozwag, Peter Oppelt, Andreas Kaufmann, Achim Schneider

**Affiliations:** ^1^ Department of Gynaecology and Gynaecological Oncology Charité University Medicine Berlin Berlin Germany; ^2^ Department of Gynaecology Kantonsspital Baselland Liestal Switzerland; ^3^ Department of Gynaecology, Obstetrics and Gynaecological Endocrinology, Kepler University Hospital Johannes Kepler University Linz Linz Austria; ^4^ Medizinisches Versorgungszentrum im Fürstenberg‐Karree Berlin (MVZ) Berlin Germany

**Keywords:** breast‐conserving surgery, lumpectomy, oncoplastic breast surgery, reduction mammoplasty, segmental mastectomy, therapeutic mammoplasty

## Abstract

**Background:**

In addition to conventional breast‐conserving surgery (BCS), oncoplastic breast surgery (OBS) is an operation technique that strives simultaneously to increase oncological safety and patient's satisfaction. It is the combination of the best‐proven techniques in plastic surgery with surgery for breast cancer. In a growing number of indications, OBS overcomes the limit of conventional BCS by allowing larger resection volumes while avoiding deformities. The aim of our retrospective study (2012–2014) was to compare oncological outcomes of OBS versus BCS.

**Methods:**

We compared two groups of patients with primary non‐metastatic breast tumours: group A (*n* = 291), where BCS was performed, versus group B (*n* = 52), where OBS was performed. Surgical interventions were performed in German and Swiss teaching hospital settings. The surgeon for group B had subspecialist training in OBS. We assessed outcome in term of re‐excision rates, resection margin and complications.

**Results:**

Groups were homogenous (no significant differences in terms of age, tumour size, tumour type or grade). The resection margin was larger in group B (7 mm) than in group A (3 mm). Re‐excision rate of group B (8%) was significantly lower than in group A (31%). Complication rates were comparably low in groups A and B.

**Conclusion:**

Despite the limits of retrospective design, our study confirms that OBS is safe and reduces the re‐excision rates and the need for further surgery. OBS has the potential to improve oncological care and should be more widely adopted.

## Graphical abstract

Oncoplastic breast surgery is oncologically safe, allows large resection volumes, achieving more frequently clear margins. The re‐excision rates are three times lower than with conventional surgery group. The need for additional surgery is drastically reduced.

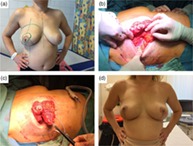

## Introduction

Conventional breast‐conserving surgery (BCS), also referred to as segmental mastectomy in medical subject heading (MeSH Unique ID: D015412), aims at removing only enough breast tissue to ensure that the margins of the resected surgical specimen are free of tumour. As a confined alternative to mastectomy, that is surgical procedure to remove one or both breasts, BCS requires adequate preoperative tumour localization and characterization. In these conditions, BCS has been revealed to be equally safe as mastectomy.

Although non‐affected tissues are conserved through BCS, most frequently the shape of the breast cannot be preserved and ultimately BCS leads to breast deformities.^1^ As a new paradigm in BCS, oncoplastic breast surgery (OBS) combines principles of oncology and plastic surgery toward achieving sound oncological and aesthetically pleasant results.^2^ Furthermore, OBS expands the indications for breast conserving allowing the resection of much larger tumours in relation to breast size. OBS is now an option for the surgical treatment of tumours larger than 4 cm and locally advanced cancers, where previously mastectomy was the only option.^3^, ^4^

OBS also allows ample margins for excision, which translate to a low rate of margin involvement and secondary therapeutic procedures.^5^ In addition, OBS prior to radiation therapy minimizes breast deformities as compared to BCS and improves breast shape.^6^, ^7^ The available evidence suggests that OBS is safe.^8^

Together, this leads to the following benefits: larger surgical margins are possible and superior aesthetic results, noteworthy in terms of breast shape. In addition, an intrinsic advantage of OBS is to avoid the need for secondary correction deformities, which result in delayed healing and poor aesthetic outcome, especially when post‐operative radiotherapy is applied.^9^

The remarkable results obtained so far with OBS hold the greatest promise and explain the increasing number of reports dedicated to the subject,^10^ some authors already considering OBS as a new standard of care,^11^ but OBS still needs to be more systematically assessed, as BCS has been.

The aim of the present retrospective study is to compare the outcome in terms of surgical resection characteristics and complications after undergoing two types of surgical intervention for primary breast cancer: conventional BCS (group A) versus OBS (group B) between January 2012 and October 2014.

## Methods

### Patient inclusion criteria

Patients presenting a non‐metastasized (local) histologically confirmed primary breast cancer (diagnosed) were included. We included patients with tumour classified as invasive carcinoma, *in‐situ* carcinoma and mixed type. Patients of all age categories were included.

### Exclusion criteria

We excluded patients presenting distant metastasis at the time of first diagnostic, patients treated by neoadjuvant therapy or mastectomy, patients with a previous occurrence of breast cancer or local recurrence.

### Patient groups and intervention

**Figure 1:**
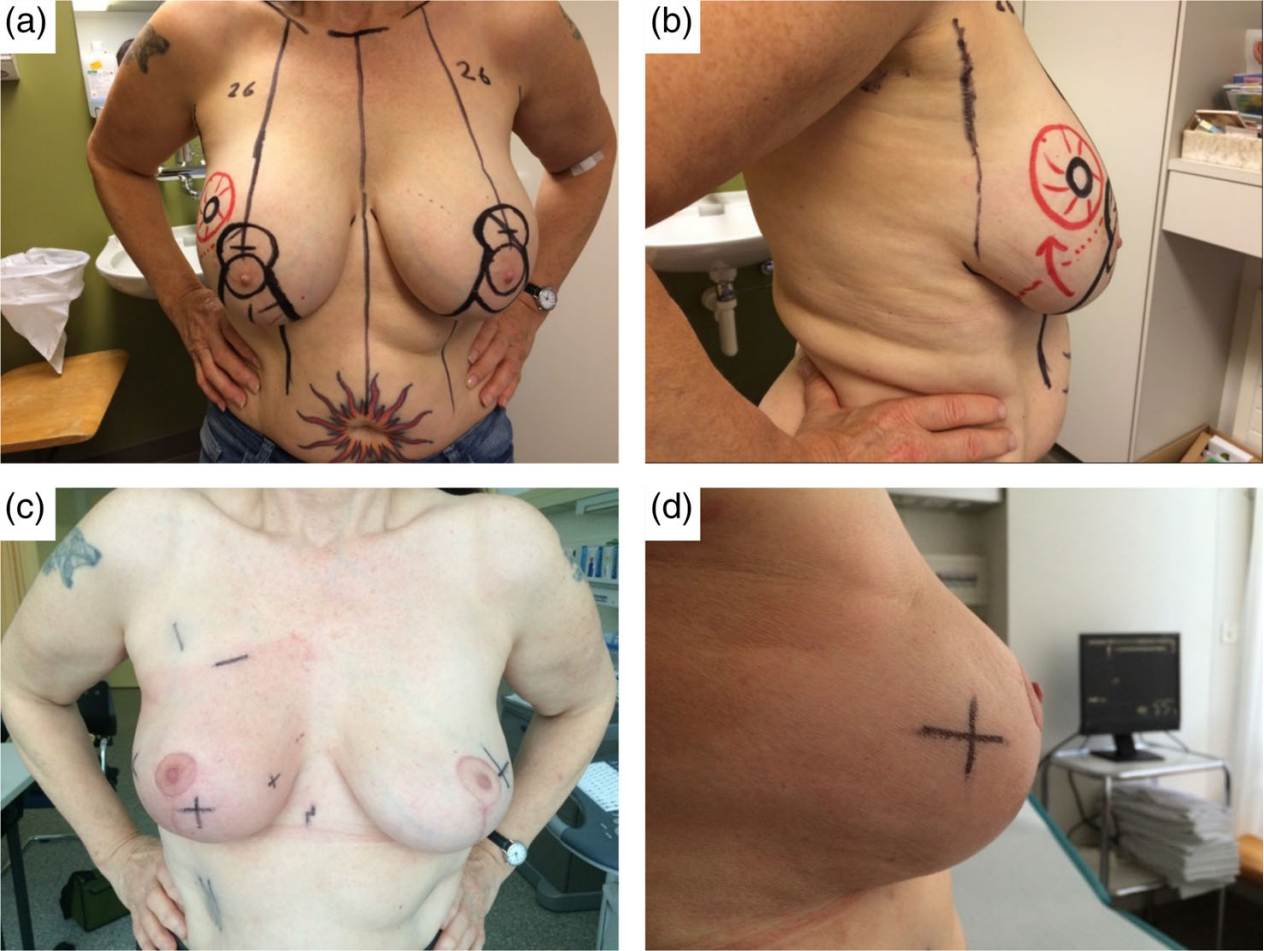
Illustrations for a 56‐year‐old patient with a 20‐mm tumour in the right upper outer quadrant and bilateral round implants. (a) and (b) Drawings for a bilateral implant removal followed by right vertical therapeutic mammoplasty (superior‐medial extended pedicle) and left vertical reduction mastopexy (supero‐medial pedicle) for symmetrization. (c) and (d) Appearance just after radiation therapy of the right breast.

**Figure 2:**
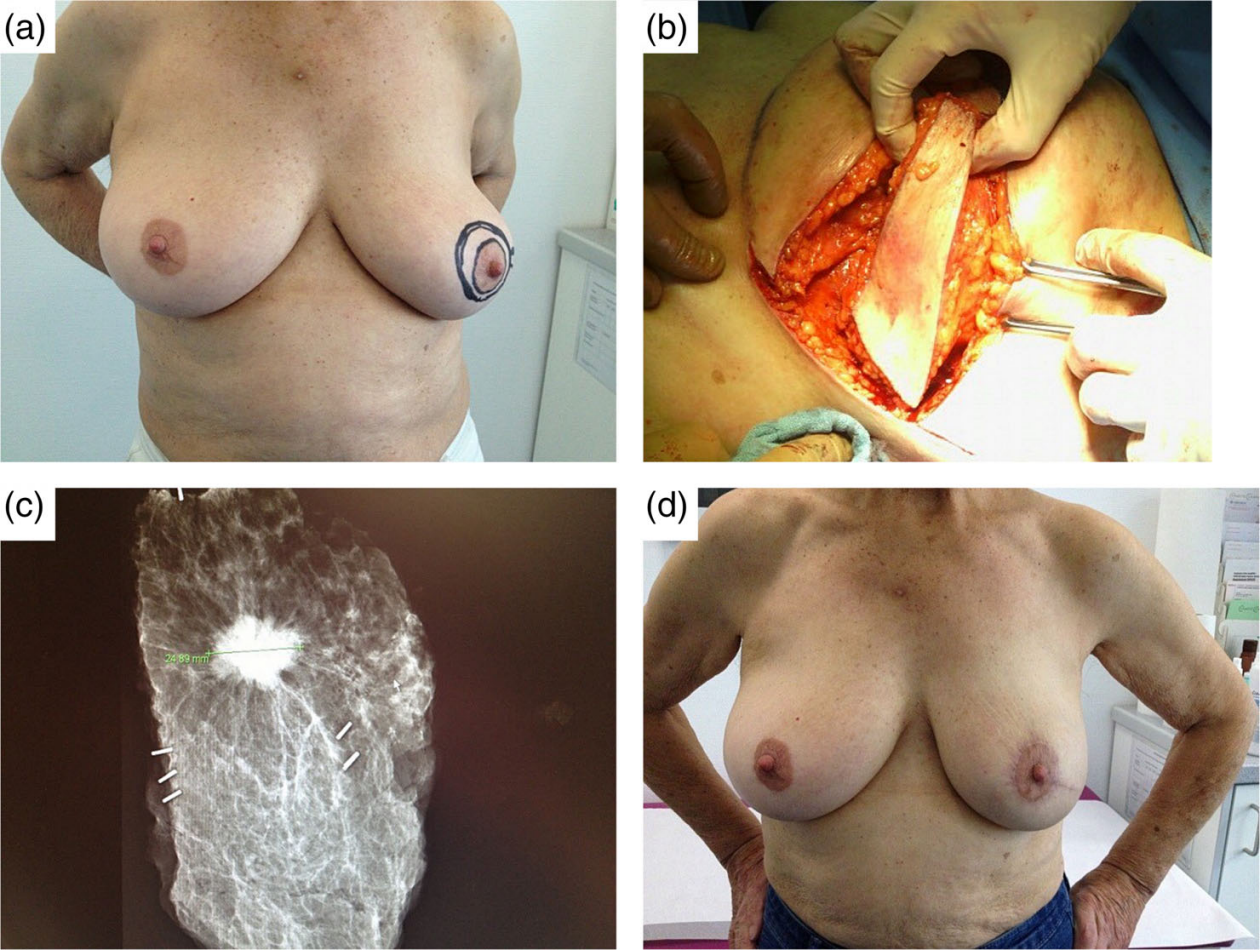
V‐mammaplasty for a 25‐mm tumour at 4 o'clock position. (a) Preoperative drawings, (b) intraoperative picture, (c) specimen radiography showing clear margins, (d) picture 3 months after radiotherapy.

**Figure 3:**
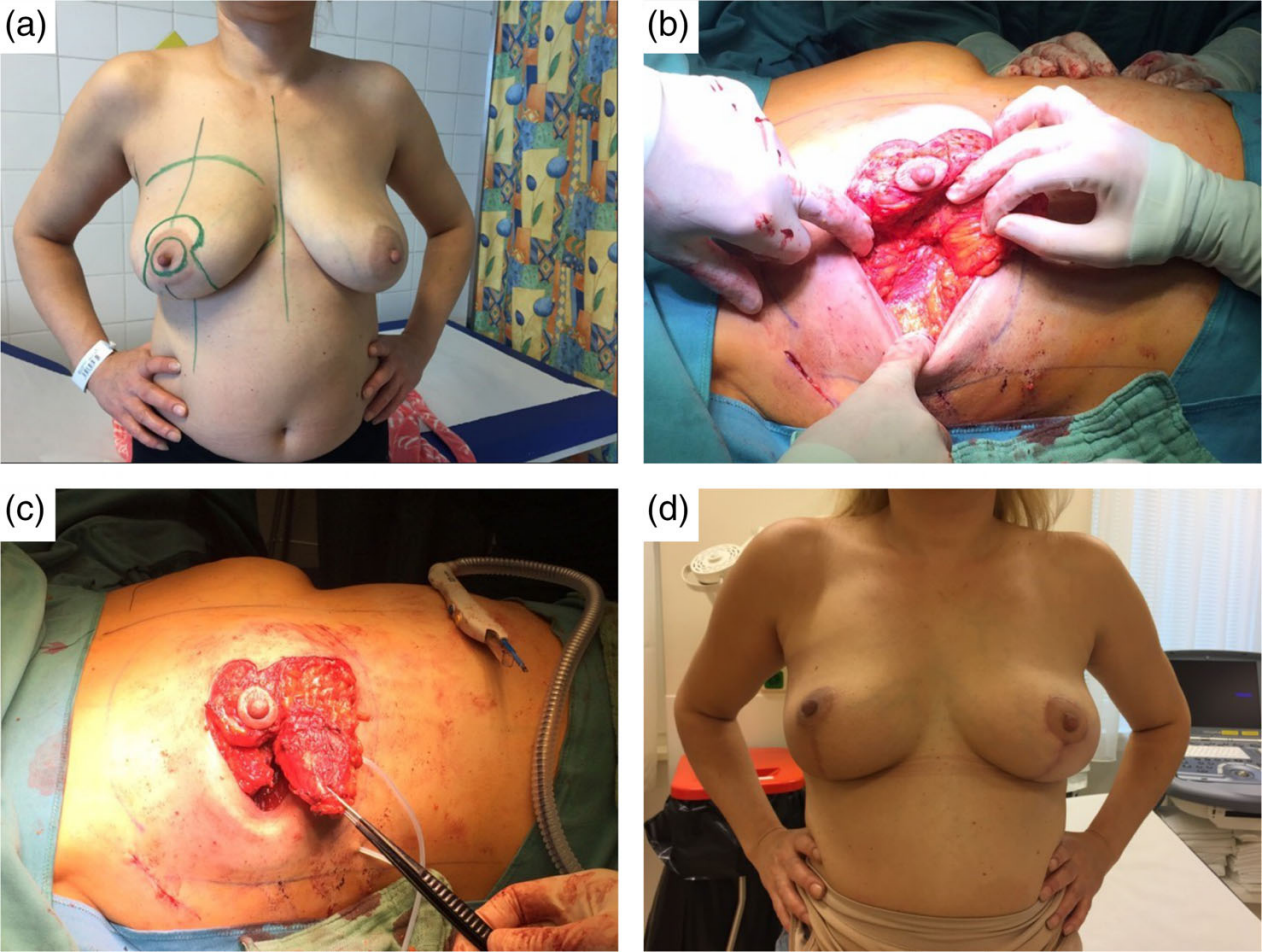
Illustration for a 46‐year‐old patient with 13‐cm ductal carcinoma *in situ* between 7 and 8 o’clock. (a) Drawings for vertical therapeutic mammoplasty, two pedicle technique supero‐medial and medial pedicle, (b) intraoperative view showing the cavity, (c) intraoperative view of supero‐medial and medial pedicles, (d) appearance after radiation therapy (6 months) and symmetrization (vertical reduction mastopexy).

Group A includes 291 patients who underwent conventional BCS consisting in standard tumour resection without reconstruction. Group B includes 52 patients who underwent OBS as primary intervention consisting of tumour resection and concomitant oncoplastic reconstruction. The techniques applied for OBS group encompass Level II techniques such as therapeutic mammoplasty,^12^ vertical mammoplasty (medial or supero‐medial pedicle;^13^ Fig. 1), inverted T‐pattern therapeutic mammoplasty,^14^ V‐mammoplasty^1^ (Fig. 2), racquet technique and round block mammoplasty.^15^ Whenever possible, vertical therapeutic mammoplasty (Fig. 3) was preferred where the breast shape is maintained by creating secondary pedicles in addition to the nipple pedicle. In practice, the pedicle selection was adapted to tumour localization. The pedicle was applied from the opposite side of the tumour.

For group A (BCS), surgical interventions involved four breast surgeons performing in a hospital setting at Charité Breast Centre, Campus Charité Mitte. For group B (OBS), all interventions were performed by one breast surgeon with subspecialization in oncoplastic‐reconstructive breast surgery at two sites: Charité Breast Centre, Campus Benjamin Franklin, in Germany and Kantonspital Baselland in Switzerland.

### Descriptive parameters

Tumour size was measured through the largest diameter (if multiple lesions, only the largest lesion was measured and reported).

We also included a description of tumour type, distinguishing invasive, mixed type and *in situ* carcinoma based on histopathological results.

We applied the pTNM staging system for all cancers in accordance with international standards. In accordance with internal protocol at Charité Berlin for breast cancer histopathological evaluation and diagnosis of mixed type tumour (e.g. with invasive and ductal carcinoma *in situ* (DCIS) components), the p‐stadium reporting was based on invasive component sizing. We did not apply further classification subdivision of T1 (no T1a, T1b and T1c sub‐grouping). For carcinoma *in situ* (devoid of invasive component), the classification Tis was applied. The lymph nodes status was reported for 84.5% of cases, noteworthy without N2 subdivision in N2a and N2b. In addition, tumour differentiation grade was assessed in most patients according to grading scale G1 to G3 (from differentiated to undifferentiated). For DCIS, we assessed the sentinel lymph nodes only for undifferentiated carcinoma (G3). Hormone status was post‐operatively evaluated through histopathological investigation of oestrogen and progesterone receptors.

### Outcome variables

We collected the following post‐operative data in order to assess the outcome.

After each primary surgical intervention, we approximated the resection volume from the dimensions of the resected specimen using the following formula:

(1) V=a×b×c

where *V* is the resection volume (cm^3^); *a*, *b* and *c* are specimen's length (cm) in the medial/lateral, superior/inferior and anterior/posterior directions, respectively. The resection specimen was marked intraoperatively with sutures to enable histological dimension assessment post‐operatively. From the histopathological evaluation of this primary resection specimen in posterior, anterior, medial and lateral, as well as superior and inferior anatomical axes, we extracted the size of resection margin as the smallest distance between tumour border and resection border. Margin infiltration status was decisive to initiating re‐excision. As outcome parameter reflecting oncological safety, we surveyed the following additional surgical interventions: number of re‐excisions if any, as well as mastectomy as final option (completion mastectomy). We monitored also the outcome in terms of operative complications by surveying necrosis, soft parenchyma hematomas and secondary wound healing defects.

### Data source and analysis

Data originate from patient dossiers, including electronic access and review of operation and pathology reports, as well as discharge letters. Data were analysed with the software SPSS 23.0 (IBM, Armonk, NY, USA). For hypothesis testing, we applied the two‐tailed *t*‐test and chi‐squared test followed by Fisher's exact test for confirmation. Statistical difference was considered significant for *P* < 0.05.

## Results

### Descriptive statistics

Group A (BCS, *n* = 291) and group B (OBS, *n* = 52) did not statistically differ for the age and the largest diameter of tumour as determined by histopathology. As such, the sub‐population corresponding to groups A and B can be considered homogeneous (see Table S1 for detailed descriptive statistics). In terms of TNM staging, we observed no significant differences between groups but worthwhile trends (low *P*‐value approaching significance level), noting that our study design excludes M1. First, with relation to T parameter, which refers to tumour size and extent, group B (OBS) tends to include fewer Tis and T1 staged tumours, that is carcinoma *in situ* and small confined tumours, but more T2 staged tumours, that is large tumours, with 42.3 versus 25.1% for group A (BCS). In addition, tumours of the overall infrequent T3 stage are mostly found in group A (BCS), with a frequency of 4.8 versus 1.9% for group B (OBS) and none of the tumours staged T4. Regarding differentiation grade, the intermediary grade G2 was the most frequent tumour differentiation grade for both groups (group A 49% and group B 50%). The second most frequent grade is G3 and the less frequent grade is G1 for both groups. Although the distributions do not statistically differ, the low *P*‐value indicates some trend to discrepancy. Indeed, group A includes proportionally fewer G3 and more G1. For both groups, the majority of sentinel lymph node biopsies performed resulted in similarly positive outcomes, without significant differences (*P* = 0.56). Most tumours considered were homogeneously positive for oestrogen receptor, 86 and 84% for groups A and B, respectively (*P* = 0.664). Most tumours were also positive for progesterone receptor with slightly different frequencies: 72 and 60% for groups A and B, respectively (*P* = 0.094).

### Outcome comparison

We compared group A (BCS) and group B (OBS) for selected outcome variables in terms of operative parameter: actual resection margins and volumes, as well as immediate outcome parameters reflecting the need for additional surgery with the re‐excision rate (Table 1). Furthermore, regarding short‐term follow‐up, we also report important complications (soft tissue haematoma, necrosis, wound healing perturbations).

**Table 1:** Outcome comparison between group A: breast‐conserving surgery (segmental mastectomy) and group B: oncoplastic breast surgery

Outcome	Group A (BCS, *n* = 291)	Group B (OBS, *n* = 52)	*P*
Resection margins (mm)			
Mean ± SEM	3.22 ± 0.19	6.98 ± 0.59	<0.001
95% CI	0.19–3.60	5.80–8.16	
*n*	291	52	
Resection volume (cm^3^)			
Mean ± SEM	79.4 ± 4.52	269.8 ± 34.76	<0.001
95% CI	70.5–88.3	200.1–339.6	
*n*	290	52	
Additional surgery: RE and mastectomy			
Rate, %	34.4	7.69	<0.001
Cases with, *n* (%)			
No RE	191 (65.6)	48 (92.3)	
One RE	91 (31.3)	4 (7.7)	<0.001
Two RE	9 (3.1)	0 (0)	
Mastectomy, *n*	33	0	<0.001

	Group A		Group B		*P*
Subgroup analysis of RE rate by cancer type					
	*n*_all_	*n*_RE_ (%)	*n*_all_	*n*_RE_ (%)	
IC	165	39 (23.6)	28	0	0.002
DCIS+I	78	40 (51.3)	16	2 (12.5)	0.005
DCIS	48	21 (43.8)	8	2 (25)	0.449
Subgroup analysis resection volume by number of RE					
	Mean ± SEM (95% CI)		Mean ± SEM (95% CI)		
No RE	79.2 ± 5.7 (67.9–90.5)		280.6 ± 37 (206–355)		<0.001
One RE	78.1 ± 7.65 (62.86–93.3)		140.75 ± 54.5 (−32.8–314.3)		
Two RE	97.1 ± 26.44 (36.14–158)		NA		

Complications	Group A, *n* (%)	Group B, *n* (%)	*P*
All types	13 (4.5)	4 (7.7)	0.283
Soft tissue haematoma	11 (3.79)	2 (3.85)	
Necrosis	1 (0.34)	2 (1.92)	
Wound healing perturbation	1 (0.34)	2 (1.92)	

BCS, breast‐conserving surgery (segmental mastectomy); CI, confidence interval; DCIS, ductal carcinoma *in situ*; DCIS+I, mixed type of DCIS with invasive component; IC, invasive carcinoma; NA, not applicable; OBS, oncoplastic breast surgery; RE, re‐excision; SEM, standard error to the mean; Tis, carcinoma *in situ.*

### Resection margin and resection specimen volume

The mean resection margin after primary intervention was significantly larger for OBS group than for BCS with 6.98 ± 0.59 and 3.22 ± 0.19 mm, respectively (*P* < 0.001). This relates to the resection volume, which was also very significantly larger in the OBS group: 269.8 ± 34.76 versus 79.4 ± 4.52 cm^3^ for groups B and A, respectively (*P* < 0.001).

### Re‐excision rate

We observed a statistically significant lower re‐excision rate in group B (OBS) than in group A (BCS), where, respectively, 34.4 and 7.7% of patient underwent re‐excision (*P* < 0.001, Table 1). No second re‐excision was revealed necessary within group B (OBS). Furthermore, no completion mastectomy was required in group B (OBS) in striking contrast to group A: 33 patients (11.3%) of which 30 were concomitant to first re‐excision and three to second re‐excision. Remarkably, subgroup analysis of the resection volume against the number of re‐excision occurrences revealed no noticeable trend in group A but an interesting one in group B (OBS), where the number of re‐excisions seems to increase when resection volume after primary intervention is lower (Table 1).

### Post‐operative complications

Overall, post‐operative complication rate was similarly low: 4.5% for group A (BCS) and 7.7% for group B (OBS), without statistically significant differences (*P* = 0.283). For both groups, soft tissue hematoma was the complication that rated most frequently with 3.79 and 3.85% for group A (BCS) and group B (OBS), respectively. Necrosis and wound healing perturbations were less frequent types of complication, both counting one patient (0.34%) or two patients (1.92%) for group A (BCS) and group B (OBS), respectively.

## Discussion

The present study relies on a retrospective design that has clear limitations, including, for example, absence of randomization and level III of clinical evidence. That said, retrospective studies do have advantages in ethical terms when evaluating surgical treatments,^16^ especially for the evaluation of new plastic surgical methods such as OBS.^1^ In the following, we discuss our results in the light of previous publications that are technically consistent with ours, emphasizing relevant discrepancies where necessary (Table S2).

OBS is less widespread than conventional BCS. Hence, as in other similar work,^17^ the contingent in group B is smaller than in group A. However, both groups revealed homogenous demographics and pathology characteristics (Table S1). The significantly longer operation duration in group B (OBS) reflects the additional time devoted to oncoplastic surgery. With the exception of a few studies involving techniques that varied slightly from ours, the results we achieved confirm statistically with the hypothesis that OBS enables larger resection volumes (on average three‐time larger) and larger resection margins than BCS^1^ (for full details refer to Table S2). Notwithstanding the debate about the significance of margins in terms of oncological safety,^18^ our results show that with OBS significantly higher resection volumes and significantly lower re‐excision rates are observed (see Table 1, compare with Table S2). The higher resection volume permitted by OBS is technically of advantage in terms of lower re‐excision rates in comparison to BCS. Indeed, our results even tend to suggest that increasing resection volumes as enabled by OBS may directly reduce the need for re‐excision (Table S1). Furthermore, subgroup analysis by cancer type (with Bonferroni correction) reveals that OBS is particularly efficient in the surgical treatment of invasive carcinoma and mixed type tumours (invasive carcinoma mixed with DCIS). These important observations warrant further study for confirmation.

We did not observe any significant difference in terms of surgical complications, and post analysis of our data revealed an association only with operation duration (data not shown). Some authors reasonably raised concerns about a potential increase in the risk of fat necrosis in OBS.^8^ Although our data do not show a strong association of OBS with skin necrosis, we have taken the precaution of reporting the tendency toward a slightly higher frequency of necrosis and wound healing perturbations with OBS. Unfortunately, our study design did not measure the extent of skin‐undermining and therefore cannot assess directly such hypotheses. Post‐analysis of our data revealed a possible relation between operation duration and post‐operative complication rates for both groups (for other parameters, including age, adjuvant therapy, tumour type and stage, hormonal status adjuvant therapies, no relation was observed, data not shown). To address more specifically the relation of skin necrosis to operative parameters, future studies involving OBS should include both operation duration and an evaluation of the extent of skin undermining. In any case, we advise that for BCS or OBS special attention be paid to all adapted measures limiting post‐operative complications. Although a detailed discussion of this is beyond the scope of this article, interested readers may refer to dedicated guidelines and reports.^19^, ^20^, ^21^, ^22^, ^23^, ^24^, ^25^

In conclusion, our results confirm that OBS is safe and reduces the re‐excision rates and the need for further surgery. Moreover, OBS permits extensive resections of breast parenchyma for treatment of breast cancer, achieves clear tumour margins, and avoids the need for further surgery – a factor which entails additional emotional burden for the patient. Although aesthetic satisfaction is another critical parameter for patients, unfortunately it was not practical in the present study to collect data on patient‐reported outcome. This is a limit of the present work that should be addressed in further study.

We believe that OBS should be an established option in the treatment of breast cancer and that it should be a standard of care. With appropriate training, this technique could indeed be safely introduced in a breast cancer service. Subspecialist training in OBS, similar to that available in UK and Australia, where since 2005 such fellowships exist, should be available in the German speaking countries.

## Conflicts of interest

None declared.

## Supporting information


**Table S1.** Descriptive statistics for the two groups investigated, group A: breast‐conserving surgery (segmental mastectomy) and group B: oncoplastic surgery.

**Table S2.** A summary of previous oncoplastic breast conserving studies.
