# Supplementary material for: Oncoplastic breast surgery versus conventional breast‐conserving surgery: a comparative retrospective study
Source: ANZ J Surg. 2019 Apr 16;89(10):1236–41. doi: 10.1111/ans.15245 (PMC6849881; doi:10.1111/ans.15245)
Supplement: Supplementary file 1 — Table S1. Descriptive statistics for the two groups investigated, group A: breast‐conserving surgery (segmental mastectomy) and group B: oncoplastic surgery. [file ANS-89-1236-s001.docx]

# Table S1: Descriptive statistics for the two groups investigated, Group A: breast-conserving surgery (segmental mastectomy) and Group B: oncoplastic surgery

| **Group** | **A** | **B** |  |
| --- | --- | --- | --- |
| n patient | 291 | 52 |  |
| **Descriptive statistics** | **Mean ± SEM** | **Mean ±SEM** | **p** |
| age (years) | 59 ± 11 | 62 ± 12 | 0.122 |
| surgery duration (min) | 83 ± 34 | 150 ± 36 | < 0.001 |
| largest tumour diameter (mm) | 25 ±18 | 24 ± 12 | 0.729 |
| **Tumour classification** | **n (%)** | **n (%)** | **p** |
| Invasive carcinoma (IC) | 165 (57%) | 28 (54%) |  |
| mixed type (DCIS+I) | 78 (27%) | 16 (31%) |  |
| DCIS | 48 (16%) | 8 (15%) |  |
| *Total* | *291 (100%)* | *52 (100%)* | *0.840* |
| **TNM staging*** | **n (%)** | **n (%)** | **p** |
| Tis | 48 (16.5%) | 8 (15.4%) |  |
| T1 | 156 (53.6%) | 21 (40.4%) |  |
| T2 | 73 (25.1%) | 22 (42.3%) |  |
| T3 | 14 (4.8%) | 1 (1.9%) |  |
| *Total* | *291 (100%)* | *52 (100%)* | *0.069* |
| N0 | 192 (79.0%) | 38 (80.85%) |  |
| N1 | 41 (16.9%) | 4 (8.51%) |  |
| N2 | 6 (2.5%) | 4 (8.51%) |  |
| N3 | 4 (1.6%) | 0 (0.00%) |  |
| *Total* | *243 (100%)* | *47 (100%)* | *0.079* |
| G1 | 66 (22.7%) | 5 (9.6%) |  |
| G2 | 142 (48.8%) | 26 (50.0%) |  |
| G3 | 83 (28.5%) | 21 (40.4%) |  |
| *Total* | *291 (100%)* | *52 (100%)* | *0.058* |
| **sentinel lymph node biopsies** | **n** **(%)** | **n (%)** | **p** |
| negative | 190 (79.2%) | 41 (83.7%) |  |
| positive | 50 (20.8%) | 8 (16.3%) |  |
| *Total* | *240 (100%)* | *49 (100%)* | *0.560* |
| **Hormone receptors** | **n (%)** | **n (%)** | **p** |
| ER+ | 248 (86%) | 42 (84%) | 0.664 |
| PR+ | 207 (72%) | 30 (60%) | 0.094 |

**Legend (Table S1):**

Abbreviations: Tis: carcinoma in situ, IC: invasive carcinoma, DCIS: Ductal carcinoma in situ, DCIS+I: mixed type of ductal carcinoma in situ with invasive component, BCS: standard breast-conserving surgery (segmental mastectomy), OBS: oncoplastic breast surgery, SEM: Standard Error to the Mean. * note that all cases are M0 since metastasized cancers (M1) were excluded in study design, no T1a, T1b and T1c sub-grouping was applied and G4 was not included in grading.
