# Supplementary material for: Oncoplastic breast surgery versus conventional breast‐conserving surgery: a comparative retrospective study
Source: ANZ J Surg. 2019 Apr 16;89(10):1236–41. doi: 10.1111/ans.15245 (PMC6849881; doi:10.1111/ans.15245)
Supplement: Supplementary file 2 — Table S2. A summary of previous oncoplastic breast conserving studies. [file ANS-89-1236-s002.docx]

Table S2: A summary of previous oncoplastic breast conserving studies

|  | | Operations | Positive margins | |
| --- | --- | --- | --- | --- |
| Year | Authors | n | n | Percentage |
| 2003 | Clough *et al.*^1^ | 101 | 11 | 11 % |
| 2005 | Kaur *et al.* ^2^ | 30 | 5 | 17 % |
| 2005 | McCulley *et al.* ^3^ | 50 | 4 | 8 % |
| 2007 | Retjens *et al.* ^4^ | 148 | 13 | 9 % |
| 2007 | Giacalone *et al.* ^5^ | 31 | 3 | 10 % |
| 2010 | Fitoussi *et al.* ^6^ | 540 | 102 | 19 % |
| 2013 | Down *et al.* ^7^ | 37 | 2 | 5 % |
| 2013 | Schaverien *et al.* ^8^ | 48 | 2 | 4 % |
| 2013 | Hamdi *et al.* ^9^ | 119 | 3 | 3 % |
| 2014 | Losken *et al.* ^10^ | 83 | 20 | 24 % |
| 2015 | Clough *et al.* ^11^ | 272 | 33 | 12 % |
| 2016 | De Lorenzi *et al.* ^12^ | 454 | 13 | 3 % |

1 Clough KB, Lewis JS, Couturaud B, Fitoussi A, Nos C, Falcou M-C. Oncoplastic Techniques Allow Extensive Resections for Breast-Conserving Therapy of Breast Carcinomas. *Ann Surg* 2003; **237**: 26–34.

2 Kaur N, Petit J-Y, Rietjens M, Maffini F, Luini A, Gatti G *et al.* Comparative study of surgical margins in oncoplastic surgery and quadrantectomy in breast cancer. *Ann Surg Oncol* 2005; **12**: 539–545.

3 McCulley SJ, Macmillan RD. Therapeutic mammaplasty - analysis of 50 consecutive cases. *Br J Plast Surg* 2005; **58**: 902–907.

4 Rietjens M, Urban CA, Rey PC, Mazzarol G, Maisonneuve P, Garusi C *et al.* Long-term oncological results of breast conservative treatment with oncoplastic surgery. *Breast* 2007; **16**: 387–395.

5 Giacalone P-L, Roger P, Dubon O, El Gareh N, Rihaoui S, Taourel P *et al.* Comparative study of the accuracy of breast resection in oncoplastic surgery and quadrantectomy in breast cancer. *Ann Surg Oncol* 2007; **14**: 605–614.

6 Fitoussi AD, Berry MG, Famà F, Falcou M-C, Curnier A, Couturaud B *et al.* Oncoplastic breast surgery for cancer: analysis of 540 consecutive cases [outcomes article]. *Plast Reconstr Surg* 2010; **125**: 454–462.

7 Down SK, Jha MBBS MS MSc PK, Burger A, Hussien MI. Oncological Advantages of Oncoplastic Breast-Conserving Surgery in Treatment of Early Breast Cancer. *Breast J* 2013; **19**: 56–63.

8 Schaverien MV, Raine C, Majdak-Paredes E, Dixon JM. Therapeutic mammaplasty – Extending indications and achieving low incomplete excision rates. *Eur J Surg Oncol EJSO* 2013; **39**: 329–333.

9 Hamdi M. Oncoplastic and reconstructive surgery of the breast. *Breast* 2013; **22 Suppl 2**: S100-105.

10 Losken A, Pinell-White X, Hart AM, Freitas AM, Carlson GW, Styblo TM. The oncoplastic reduction approach to breast conservation therapy: benefits for margin control. *Aesthet Surg J* 2014; **34**: 1185–1191.

11 Clough KB, Gouveia PF, Benyahi D, Massey EJD, Russ E, Sarfati I *et al.* Positive Margins After Oncoplastic Surgery for Breast Cancer. *Ann Surg Oncol* 2015; **22**: 4247–4253.

12 De Lorenzi F, Hubner G, Rotmensz N, Bagnardi V, Loschi P, Maisonneuve P *et al.* Oncological results of oncoplastic breast-conserving surgery: Long term follow-up of a large series at a single institution: A matched-cohort analysis. *Eur J Surg Oncol* 2016; **42**: 71–77.
